# An examination of the growing US undergraduate public health movement

**DOI:** 10.1186/s40985-016-0048-x

**Published:** 2017-02-01

**Authors:** Beth Resnick, Suzanne Selig, Richard Riegelman

**Affiliations:** 1 University of Illinois Chicago School of Public Health, 1603 West Taylor Street, Chicago, IL 60612 USA; 2 Department of Health Policy & Management, Johns Hopkins Bloomberg School of Public Health, 624 N. Broadway, Rm. 457, Baltimore, MD 21205 USA; 3 University of Michigan-Flint, 303 E. Kearsley Street, Flint, MI 48502 USA; 4 Milken Institute School of Public Health, George Washington University, 950 New Hampshire Avenue, NW Washington, DC 20052 USA

**Keywords:** Undergraduate Education, Undergraduate Program, Public Health Education, Public Health Workforce, Undergraduate Major

## Abstract

**Objectives:**

With over 10,900 public health bachelor’s degree graduates conferred in 2015, public health undergraduate education in the USA has become mainstream. However, with the recent establishment of a majority of the programs, the impact of the undergraduate programs remains largely unknown. This study examines a sample of undergraduate programs in public health to further elucidate the undergraduate landscape.

**Methods:**

Semi-structured interviews and a review of program websites from a sample of 39 institutions across the USA with undergraduate majors labeled as public health were conducted in 2015 to examine program content and operations.

**Results:**

Findings from the 39 programs reviewed demonstrated growing and diverse undergraduate public health programs rapidly evolving. While program enrollments, infrastructure, and curriculum varied among the individual programs, collectively, findings indicated increasing numbers of undergraduate students gaining knowledge and experience in matters related to the health of societies locally, nationally, and globally.

**Conclusions:**

Study findings suggest it is an opportune time for the field to offer guidance, support, and vision to these burgeoning undergraduate programs. Such engagement offers opportunities to advance the programs as well as increase the number of students attuned to societal health in whatever life roles they assume.

## Background

Historically, formal public health education in the USA was centered at the graduate level. Although some undergraduate programs in public health were established in the twentieth century, the majority have emerged over the last decade [1]. The rise of this undergraduate phenomenon in the USA aligns with both the Institute of Medicine’s (IOM) 2003 call for undergraduate education in public health [2] and the upsurge of undergraduate student interest and engagement in public health, global health, and related disciplines over the last decade [3].

In 2015, there were over 10,900 bachelor’s degrees conferred in public health and its related disciplines, compared to 1,430 in 2003 [4]. In addition to undergraduate majors, there has been a rise in public health concentrations, minors, and general education courses, as well as public health-focused summer programs, internships, international experiences, clubs, and other extra-curricular activities [5].

In alignment with this growth in undergraduate public health education, the Association of Schools and Programs of Public Health (ASPPH) and other national public health organizations have broadened their attention beyond graduate education to advance and engage undergraduate programs. Such efforts include the development of the Undergraduate Public Health Learning Outcomes in 2011 [6] and the Recommended Critical Component Elements of an Undergraduate Major in Public Health in 2013 [7, 8]. In 2011, an annual Undergraduate Public Health and Global Health summit was initiated; in 2016, there were over 200 summit participants from nearly 150 institutions located in 42 states and 7 countries [9]. Building on the success of the summits and continued growth in undergraduate programs, in 2015, ASPPH established an undergraduate network for public health and global health education for institutions offering undergraduate majors in public health and/or global health to collectively identify key issues and advance public health undergraduate education. As of 2016, this network has over 165 institutions participating [5].

Furthermore, the accrediting body for public health schools and programs, the Council on Education for Public Health (CEPH), has expanded accreditation to bachelor’s degree programs. Accreditation was initiated in 2003 and available only to undergraduate programs affiliated with accredited schools of public health. In 2008, accreditation for undergraduate public health programs was expanded to include programs affiliated with accredited public health programs. Lastly, in 2013, accreditation was made available to standalone baccalaureate programs. As the accreditation process takes nearly 3 years to complete, accreditation of standalone undergraduate programs is still in its infancy. As of summer 2016, there were nearly 90 accredited undergraduate programs in public health, four as standalone programs, the remaining affiliated with accredited schools and programs, and over 20 undergraduate public health accreditation applications in process [10, 11].

In the workforce realm, the Certified in Public Health (CPH) examination eligibility criteria was expanded in 2015 to accept exam candidates with bachelor’s degrees in any discipline along with core public health education and 5 years relevant work experience [12]. As such, to become certified in public health, candidates must demonstrate core public health knowledge upon experience and/or education.

## Undergraduate public health education as part of the continuum of public health education

In 2015, the Association of Schools and Programs of Public Health (ASPPH) published a series of reports as part of the Framing the Future: The Second Hundred Years of Public Health Education [13]. These reports lay out a vision for public health education across the post-secondary continuum, from doctoral to master’s to bachelor’s and associate degrees. As public health education in the USA had formally been centered at the graduate level, these reports raised awareness of and attention to public health education at the undergraduate level within the academic community, as well as public health professionals.

## Opportunities to influence undergraduate education

Undergraduate education in the USA is evolving and expanding access to non-traditional and international students. Online learning including hybrid/blended courses are rapidly developing, and e-portfolios and other new methods of evaluation, and an increased emphasis on competencies/learning outcomes and experiential learning, as well as joint baccalaureate and master’s degree programs are influencing all aspects of undergraduate education. Public health education is no exception; its intrinsic interdisciplinary and global underpinnings are well suited for such educational innovations.

For example, in the USA, general education, defined as broad coursework taken by all students in the first and second years of a bachelor’s degree, in some institutions is evolving into integrative general education or integrative liberal education. The Association of American Colleges and Universities (AAC&U) has led a national effort to reframe general education to extend throughout the bachelor’s degree and integrating with a major [14]. The AAC&U Liberal Education and America’s Promise (LEAP) initiative has produced a model for integrative liberal education based on LEAP Essential Learning Outcomes [15]. The AAC&U evidence-based thinking based on epidemiology principles forms the core of the AAC&U’s Scientific Thinking and Integrative Reasoning Skills (STIRS) framework, which is being developed as an exemplar of integrative liberal education through the STIRS project [16]. Such work provides a general education platform to foster awareness of and skillsets to address global public health challenges to undergraduate students in institutions across the USA. Furthermore, the STIRS project serves as a leading example of how public health in general and epidemiology in particular can contribute to the education of all undergraduates.

Thus, the changing undergraduate landscape offers increasing opportunity to advance undergraduate public health education and engage students in matters of societal health in whatever life roles graduates assume. However, given the recent emergence of undergraduate public health, impacts and opportunities from this phenomenon remain uncertain. Thus, research is needed to explore the undergraduate public health landscape. This research aims to begin to address this knowledge gap.

## Research study

This study examines US undergraduate curricula in public health through review of a sample of undergraduate programs in public health. This study is presented as the start of an ongoing need to further understand these undergraduate programs for the betterment of the field and ultimately efforts to improve the public’s health.

## Methods

Semi-structured qualitative interviews with representatives from institutions offering a baccalaureate degree in public health were conducted. The study sample was derived from a query of the National Center for Education Statistics (NCES) College Navigator database of 4-year institutions offering baccalaureate majors in general public health [17]. Websites from institutions identified in the College Navigator query were reviewed to confirm an offering of a baccalaureate degree labeled as public health. Degrees titled anything other than public health (e.g., community health and health education) were excluded from the study. The contact person listed on the website for each identified public health program was emailed an invitation to participate in a semi-structured telephone interview to discuss the content and operations of their undergraduate program in public health.

For any eligible public health programs without a contact person listed on the website, program directories were searched for the undergraduate public health program director or coordinator and then the identified individual was emailed an invitation to participate. In addition to the interviews, program websites were reviewed for additional programmatic information. Each interview was recorded and professionally transcribed. Interview and other program documentation were coded and analyzed using NVivo software and Microsoft Excel.

This research was approved by the University of Illinois Chicago institutional review board and deemed as non-human subjects research. All interviews were voluntary, and there were no incentives offered to participate.

## Results

Two hundred and seventy-three institutions were identified in the College Navigator database from a query for baccalaureate degrees in “public health.” Website confirmations of degree offerings at these 273 institutions found 74 institutions met the study inclusion criteria of offering a bachelor’s degree titled “public health.” Two of the eligible institutions were eliminated due to the principal investigator’s affiliation, resulting in a final total of 72 eligible institutions for the study. Representatives from all 72 degree programs were emailed up to 2 invitations to participate in a semi-structured interview from June to September of 2015. Representatives from 39 (54%) of the 72 eligible institutions participated in an interview. Characteristics of these 72 institutions and the undergraduate programs are outlined below and summarized in Table 1.

**Table 1:** Characteristics of the study programs. Study sample *n* = 39

	Academic home			Total
Institutional and program characteristics	School of health professions *n* = 18	School of public health *n* = 15	College of arts and sciences *n* = 6	
Establishment of the PH major				
Before 2000	1	2	1	4
2000–2010	6	6	2	14
2011–2015	11	7	3	21
Geographic location				
South	3	7	3	13
West	5	5	1	11
Northeast	6	3	1	10
Midwest	5	0	0	5
Institution size				
<5000	9	0	5	14
5000–20,000	6	8	1	15
>20,000	3	7	0	10
Application into the major				
Application required	3	11	1	15
Total no. of students in the major				
<50	6	1	1	8
50–149	5	5	3	13
150–499	6	5	2	13
>500	1	4	0	5
Degree offerings				
BS only	16	12	0	28
BA only	1	1	2	4
BS or BA	1	2	4	7
Minor	6	11	5	22
MPH	7	15	0	22
Internship				
Internship required	14	5	3	22

## Study sample

### Interviewees

The majority of study interviewees were undergraduate in public health program directors (24) followed by academic advisors (8) and core program faculty (7). The majority of interviewees were female (29) and overwhelmingly Caucasian >75%. Although age of the interviewees was not asked, the academic rank of the faculty interviewees indicated most were still advancing in their careers, with the most common respondent rank of associate professor (15), followed by professor (7), assistant professor (6), and instructor/special faculty (2). All interviews were conducted from June to August 2015. Thirty of the respondents had graduate-level training in public health. Of the respondents without public health training, the most common training was in nursing, followed by doctoral degrees in the social sciences.

### Academic home

Programs in the study sample were characterized into three types of academic homes: programs housed within a school of public health, college of arts and sciences, or a school or department of health professions, and were categorized accordingly in Table 1. Schools of health professions housed the largest number of programs in the study sample (18) followed by schools of public health (15) and colleges of arts and sciences (6).

### Program establishment

In alignment with the national growth of undergraduate programs in public health, the overwhelming majority of programs in the study sample (35) were established since 2000 with the majority (22) established from 2011 to 2015 [1].

When asked what spurred the establishment of the public health major, respondents most frequently noted rising student interest in public health and that the major aligned with their school/department’s mission and existing educational offerings. Additionally, respondents frequently cited wanting to stay “current” in educational offerings as a contributing factor in establishment of the public health major. Staying “current” was most often noted with regard to the establishment of a public health major at other undergraduate institutions. A few respondents (~4) cited an external push to establish the major. In private institutions, respondents reported this push emanated from alumni or board members whereas in public institutions, the push was reported from policy makers or state education leaders.

### Geographic location

Geographically, the largest proportion of institutions in the study sample were located in the South (13): Alabama (1), Arkansas (1), Florida (1), Georgia (2), Kentucky (1), Louisiana (1), Oklahoma (1), Tennessee (1), Texas (1), Washington, DC (2), and West Virginia (1); followed by the West (11): Arizona (2), California (3), Colorado (1), Hawaii (1) Oregon (3), and Washington (1); and the Northeast (10): New Hampshire (1), Massachusetts (3), New York (2), Pennsylvania (2), New Jersey (1), and Connecticut (1). The smallest number of institutions in the sample was located in the Midwest (5): Illinois (2), Ohio (2), and Minnesota (1). The geographic locations of the sample institutions align somewhat with national data on all undergraduate public health programs from the National Center for Education Statistics (NCES) that indicated the highest percentage of institutions with undergraduate programs in public health in the South (35%); however, nationally, the next largest proportion of institutions with undergraduate programs in public health were in the Midwest (26%) and the smallest proportion (18%) in the West [1].

### Institution size

With regard to the size of the institution’s total undergraduate student body, the interview sample again comported with the national undergraduate public health landscape [1]. The largest proportion of institutions both in the nation and in the study sample of undergraduate programs in public health were housed in the largest institutions with greater than 20,000 students (15 in the study sample) and in the smallest institutions with less than 5000 students (14 in the study sample). The fewest proportion of institutions in the nation and in the sample were in the middle of these size ranges (5000–20,000 students) (9 in the study sample).

Programs in the study sample housed in schools of public health (15) were all located in large research universities, predominately public (12) with student bodies >10,000, whereas programs housed in schools of health professions (18) were located in the widest range of institution sizes, ranging from <5000 (9), between 5 and 20,000 (6), and over 20,000 (3), and slightly more than half were in private institutions (10). Lastly, programs in the study sample housed in colleges of arts and sciences (6) were mostly in the smallest and private institutions (5), with only one institution with over 5000 students.

### Program enrollments

Across the study sample, respondents reported total student enrollment in the public heath major ranged from 18 to 3200 students. The largest enrollments were reported in programs housed in schools of public health (range 48–3200, median 157) followed by programs housed in schools of health professions (range 18–950, median 54) and programs in colleges of arts and sciences with the smallest reported enrollments in the major (range 35–175, median 74).

Interview respondents across the sample reported rapid enrollment growth over the last 2 years (2013–2015), which is consistent with the national undergraduate degree conferral data [1]. Furthermore, three respondents specifically noted that the name change of their majors to public health from community health or health education resulted in marked increases in student enrollments (>20%). When respondents were asked to identify the underlying reasons for the increasing popularity of the major, collectively they expressed uncertainty. However, several themes emerged from these discussions that are outlined below.

One sentiment that emerged from the discussions with respondents, which comports with findings from undergraduate education overall, was the perception of undergraduate education as an “investment” and families seeking the best return on their investment [18]. As such, selection of an undergraduate major often went beyond intellectual interest to focus on value of the major. In this regard, respondents highlighted the fact that students, and in particular their parents, perceive public health as a “valuable degree” leading to employment or serving as a pre-professional pathway to medical or allied health careers.

Respondents also associated this “return on investment” sentiment with an increase in undergraduate double majors and minors. Again, this study finding of increasing double majors and minors corroborated with national trends in undergraduate education overall [19]. Respondents emphasized that public health, at its core, is interdisciplinary; thus, it is well positioned as a dual major or a minor. One respondent, from a liberal arts college, highlighted that they had public health students with a wide range of double majors, including Spanish, biology, biochemistry, psychology, environmental and sustainability studies, anthropology, chemistry, economics, French, theology, math, and religion. Twenty-two programs in the sample offered a public health minor with about half of these respondents noting that the minor was a recent addition to their program (within the last 2–3 years).

Another factor respondents cited as a possible predicator of the increasing popularity of the major was increased student interest in matters related to health and wellness. Respondents surmised that perhaps this increased interest was in part related to national attention on health care reform efforts and health disparities. Additionally, in line with the “value of educational investment” notion described above, respondents noted that students and their parents viewed the health arena positively in terms of career opportunities.

Another point raised by respondents in relation to popularity of the major was public health’s “do good” appeal. Many of the students in the major were described by respondents as “value driven,” embracing social justice and a desire to work with vulnerable populations to address unmet community needs. This appeal was emphasized particularly from respondents in programs and schools with strong social justice underpinnings. Additionally, respondents emphasized that these “value-driven” students were drawn to service-learning opportunities in local communities and globally.

Lastly, respondents from institutions offering nursing and other allied health degrees with restricted enrollments noted public health’s appeal as an alternative major for students not accepted into the restricted majors. Public health as an alternative option was also noted by respondents in institutions with students struggling in the pre-medical curriculum.

In sum, there was consensus among respondents that enrollments in the public health major are increasing. Discussion with respondents about this phenomenon revealed a range of possible predicators that varied among the individual institutions and reflected some trends in undergraduate education overall.

### Admission criteria

The majority of programs in the sample required students to declare their major by the end of their sophomore year. If students were in good academic standing, as per their institution’s requirements, they could declare public health as their major. However, some schools in the sample (15) had an application process into the public health major and four respondents indicated a cap on enrollments in the major.

Respondents from programs in schools of public health were the only ones to report enrollment caps (4) and were the most likely to report a competitive application process into the major (10). Three respondents from programs in schools of health professions reported requiring an application to enter the major; however, none of these respondents indicated a competitive application process. Only one respondent from a program in a school of arts and sciences reported an application process to enter the major, and this respondent did not indicate that it was a competitive process.

### Students

For the most part, respondents indicated that their programs mostly catered to full-time residential students; however, four respondents indicated having part-time students and online courses available. All but two programs employed a non-cohort model for the public health major requirements. The two institutions using a cohort model required students enrolled in the major to take a core set of courses in a defined sequence. Additionally, although three respondents indicated an interest in working with community colleges to foster transfers from associate to bachelor’s degrees in public health, only two respondents mentioned formal relationships and institutionalized efforts to transition students from community college into undergraduate majors at their institutions.

All respondents across the sample reported student bodies that were overwhelmingly female ~75–80% and, in most cases, reported that students in the public health major were a bit more diverse than the rest of the student body across the institution, across a range of incomes, ethnicities, and nationalities. However, a few (3) programs housed in schools of public health, all with competitive applications into the major, indicated that students in the public health major were less diverse than the rest of the student body at the institution.

Conversely, four respondents from institutions with very diverse student populations emphasized public health’s intrinsic appeal to such populations, in particular noting that students from immigrant families “get public health” in a personal way and tend to gravitate towards internships and employment that engages them in the support of the health of their communities. Veterans and disabled populations were also noted by some respondents as students that were drawn to public health based on their own experiences.

A number of respondents reflected that students’ diverse economic, cultural, geographic, and life experiences provided a rich and robust perspective to address today’s multi-faceted and complex public health problems. For example, one respondent highlighted the benefit of having students from malaria countries share their personal experiences with the disease with the class.

In sum, students in the sample programs were reported to be mostly full-time residential college students that were overwhelmingly female and for the majority of the programs more diverse than the institution’s overall student body.

### Degree offerings

The Bachelor of Science degree was the most commonly offered degree within the sample programs (33), seven programs also offered an option for a BA, and four programs offered only the BA degree. The option for a BA degree was available in all programs housed in schools of arts and sciences (6).

As noted above, 22 programs in the study sample reported offering a minor in public health. The credits required for a minor ranged from 15 to 29 with 18 credits (9 programs) being the most common requirement for a minor. One respondent highlighted that the public health minor was an ideal opportunity to engage more students in public health content, as well as a source of additional tuition revenue, without the additional administrative and advising burdens of students majoring in public health.

Twenty-two programs in the study sample offered master’s in public health (MPH) degrees including all programs housed in a school of public health (15), seven of the programs in schools of health professions, and none of the programs in colleges of arts and sciences.

A few programs (4) recently initiated or were in the process of establishing an accelerated bachelor’s to master’s in public health degrees. This finding comports with trends in undergraduate education overall towards such accelerated degrees [20]. One respondent reported the initiation of a new accelerated bachelor’s to master’s of public health degree at their institution in collaboration with a graduate institution in another region of the country. In this arrangement, the student will receive the bachelor’s degree in a residential on-campus program and the master’s degree online from the other institution.

### Curriculum

Interview respondents across the sample reported an evolving curriculum with approximately (18) interviewees noting curriculum revisions completed in the last year or planned for the upcoming year. Curriculum revisions were attributed to a variety of factors but most frequently were predicated on meeting accreditation requirements and to align coursework with the expertise of faculty members new to the major. Additionally, a number of respondents also associated curriculum changes with the establishment of new concentration areas within the major, such as global health or health equity.

Furthermore, respondents discussed a number of challenges in developing the curriculum. Such challenges included limited resources and faculty available to develop and teach courses, as well as barriers due to undergraduate tuition disbursement policies. Other challenges emphasized by respondents highlighted the politics involved in setting and changing curricula requirements. Such political challenges are common across academia and are certainly not unique to the public health major. However, such political challenges are particularly difficult for newly established public health majors with faculty that tend to be younger and newer to the institution than other more established degree offerings in the school. Specific political challenges discussed with respondents included lack of control over which faculty were assigned to teach the core courses and tension among departments and faculty members in making determinations of which courses satisfy general education, public heath core, and public health elective requirements. Lastly, availability of physical classroom space and time slots available for courses were also noted by respondents as other curriculum-related challenges.

Given the evolving nature of the curriculum and varying graduation and general education requirements across institutions and academic departments, it was not feasible to complete a systematic assessment of program curricula across the study sample. Additionally, there were no discernible patterns in curricula across the various academic homes or types of degrees offered (Bachelor of Science vs. Bachelor of Arts). However, there were some curriculum consistencies noted across the study sample. All the programs included a public health curricular core, which typically was between 30 and 40 credits and elective requirements that ranged from 6 to 20 credits. Epidemiology was the only consistent course included in the core curriculum across all institutions in the sample. Nearly all programs in the sample included as either part of the pre-major requirement or within the core curriculum courses an introduction to public health, an introduction to statistical concepts, and an overview of at least one natural science. Many programs in the sample also had writing, a social science discipline, and foreign language requirements as part of the prerequisites to enter the major. Global, environmental, and behavioral health, as well as program planning and evaluation, health policy, health systems, research/quantitative methods, and health disparities, were courses frequently included in the core curriculum. Additionally, many programs included a seminar, a culminating capstone course, or internship as part of the core requirements. A range of broad course titles unique to individual institutions without accompanying syllabi, such as “health meets life” and “contemporary issues in public health,” were unable to be characterized.

Lastly, the seemingly widespread inclusion of public health 101, epidemiology 101, and global health 101 suggested the likely adoption of this three-course recommendation included in the Association for Prevention Teaching and Research (APTR) and Association for American Colleges and Universities (AACU) Educated Citizen and Public Health Curriculum Guide for Undergraduate Public Health Education [21].

### Internships

A majority of the programs in the sample (22) required an internship, and programs that did not require internships did encourage them and allowed students to receive academic credit for internship experiences. Internship requirements across the sample as credits ranged from 3 to 12 credits and as hours ranged from 100 to 400 h. Respondents collectively reported that many students completed the internship requirement on a full-time basis over the summer. In lieu of an internship, a few programs (3) required service learning or other experiential learning-based courses. Internships were most likely to be required in programs housed in schools of health professions (13) followed by schools of public health (5) and then colleges of arts and sciences (3).

Many respondents highlighted that students often obtained full-time jobs at their internship site. Health departments were the most often mentioned internship site, but a wide range of other organizations were also noted including hospitals, non-profits, other community organizations, and federal government agencies (e.g., CDC). Although there were a number of respondents that reported increasing popularity of public health study abroad opportunities or international public health trips, there was no mention by any respondents of students completing international internships.

### Challenges and opportunities

Respondents were asked to discuss particular challenges and opportunities in their programs. Findings are summarized below by academic home.

Respondents from programs housed in schools of health professions highlighted the rapid growth in their programs and the synergies of the public health major with their other allied health programs. Such collaborations offered opportunities for cross-cutting thinking that meld health care and public health strategies and approaches. Additionally, respondents noted their strong internship programs and ability to build formalized relationships with the practice community that continues to strengthen as their program alumni often transition to full-time employment at their internship sites. The challenge that these respondents most often highlighted was the perception of the public health major as the “Plan B” option for students not accepted into competitive allied health programs. However, respondents noted that this challenge, while ongoing, is beginning to lessen with growing numbers of students selecting the public health major as their first choice. Additionally, this growth in students is creating opportunities for new faculty in the public health major and a growing alumni network that can support and advance the program.

Respondents from programs housed in schools of public health highlighted the fact that their programs were embedded in schools of public health which offered many opportunities for students to engage with the school’s larger public health community (faculty, doctoral students, etc.), as well as engage in ongoing public health practice and research activities. However, the key challenge noted by these respondents was gaining the acceptance of undergraduates into a previously graduate-only-focused academic entity. Thus, developing courses and activities appropriate for undergraduates and articulation between the undergraduate and master’s programs was an ongoing challenge. Additionally, most of these respondents noted that financial, physical, and organizational arrangements between the school of public health and the undergraduate campus were often quite difficult. However, collectively, these respondents indicated that although such challenges were ongoing, they were lessening with time as the undergraduate programs became an integral part of the school, both from a cultural (undergraduate students acclimating into the school community) as well as financial (undergraduate tuition revenue becoming a major source of ongoing support for the school) perspective.

Respondents from schools of arts and sciences highlighted their program’s interdisciplinary focus grounded in a liberal arts framework. These respondents were pleased that their students were building critical thinking and analysis skills that are well suited to address complex and multi-faceted public health concerns. Challenges these respondents highlighted were the limited resources, faculty, and opportunities in small liberal arts institutions. However, these respondents highlighted the growth of the major across undergraduate institutions as opportunities for them to share best practices and curriculum and to build collaborations with other institutions (e.g., shared classes, experiential opportunities, and study abroad trips).

Thus, in sum, respondents described a range of challenges and opportunities across the different institutions and academic homes. Respondents collectively expressed interest in learning more about other undergraduate programs and expanding opportunities to share experiences and best practices. The development of such a learning community is addressed in the “Discussion and implications” section.

### Post-graduation pursuits

As many programs in the study sample were recently established, post-graduation data was not readily available. However, the respondents’ accounts of post-graduation activities were fairly consistent across the study sample. Respondents estimated about one third of graduates going on for further graduate training, including MPHs and health professions, such as medical school and allied health professions, as well as a range of other disciplines, including law, business, psychology, and social work; another third of graduates were working full time in a wide range of employment which most frequently included public health agencies, non-profits, hospitals, and insurance companies; and a third of program graduates were categorized as engaged in “other activities.” A wide range of activities were cited in “other activities,” including fellowships; service programs, such as Teach for America or international work including Peace Corps; or other global health programs.

An important distinction in reported post-graduation pursuits across the study sample was that students of lesser means were most likely to seek full employment in their home location whereas students of greater means had a broader trajectory that included graduate studies or other activities in a range of local, national, and global locations.

### Discussion and implications

Although this research was limited to a sample of 39 programs, it comported with the national data that indicates that public health is a rapidly growing major and the move to accreditation is underway both for programs affiliated with accredited schools and programs of public health and now standalone programs [1]. Additionally, reports in the literature are emerging on undergraduate public health activities from institutions across the USA that appear to correlate with this study’s findings [5].

Undergraduate education in public health offers opportunities to expand access to public health knowledge to diverse populations and employ innovative education methods. This is critical to address pervasive and multi-facetted health disparities, as well as to cultivate a public health workforce that is representative of the population it intends to serve.

Such educational opportunities include the “flipped classrooms,” where key content can be learned prior to classroom sessions using digital material. This can free up face-to-face class time to focus on interactive exercises and case studies engaging students in seeking solutions to current and emerging public health challenges. Online courses are an additional new opportunity to connect the continuum of master’s to bachelor’s degree and associate degree programs, expanding access to public health content to non-traditional students. Summer coursework and hands-on public health experiences across the USA and globally along with blended or hybrid courses are other opportunities for expanding public health education approaches and impacts to a broad range of student populations.

Additionally, the Association of American Colleges and Universities (AAC&U) is advocating integrative education throughout the bachelor’s degree. Integrative education includes “guided learning pathways” in which broad and integrative education are interdigitated with specialized education, i.e., majors throughout the bachelor’s degree [22]. AAC&U’s Scientific Thinking and Integrative Reasoning Skills (STIRS) initiative aims to make evidence-based thinking an integral part of bachelor’s degree education for all undergraduates [15]. Undergraduate public health education is well positioned to contribute to these efforts due to the inherently integrative nature of public health and its emphasis on evidence-based thinking.

The Community Colleges and Public Health report represents a new direction for public health education [23]. Community colleges typically offer associate degrees and certificate programs and aim to provide entry-level career opportunities and opportunities to transfer credits to bachelor’s degree programs. As noted in the study findings, a few undergraduate programs in public health are initiating such efforts in collaboration with community colleges.

The Community Colleges and Public Health report aims to foster the continuum of public health education extending from associate degrees to doctoral degrees. The report outlines two recommended curriculum models: (1) Public Health: Generalist and Specialization, and (2) Health Navigator. The Public Health: Generalist and Specialization model is designed for transfer to bachelor’s degree program in general public health, health education, health administration, and environmental health.

Such an educational infrastructure for public health throughout the USA offers promise to expand public health professional pathways, in particular to diverse and non-traditional students underrepresented in the current public health workforce. Additionally, the growing undergraduate educational infrastructure offers new opportunities for continuing education to those already in the public health workforce. Such a role may be fulfilled by community colleges particularly for current employees without any background in public health.

However, the emergence of undergraduate education in public health underscores increasing needs for articulation between public health master’s and bachelor’s degrees. Moreover, it is critical to consider such questions as “will public health graduates be employed, and if so, where?” In addressing such questions, traditional perceptions in the USA of the “public health workforce” as professionals working in governmental public health agencies need to be expanded to other organizations. Interdisciplinary pathways to employ public heath knowledge and skills, such as urban planning, transportation, and law need to be embraced. Ultimately, to a large extent, employment opportunities for graduates of bachelors programs in public health rests with the local economy; the health status and challenges faced by the population; the educational level of the existing labor force; the policies of the respective governmental entities that determine what services will be provided and by whom, and what services will be covered by insurance policies; and the mobility of the graduates.

Such post-graduation employment questions as well as other key opportunities and challenges for undergraduate education in public health are ripe for discussion within the growing national undergraduate public health network in the USA. Such a network, already in existence via ASPPH and other leading public health organizations and supported by this study’s interview respondents, could be expanded and engagement increased with the practice community to cultivate a more robust and dynamic “learning community.” Such an approach could stimulate critical thinking, active dialog, and assessments across programs and in collaboration with the public health professional community as to how best to prepare undergraduate students to address identified public health needs and disparities locally, nationally, and globally. Such efforts should be done collectively as a movement building on the strengths and diversity of institutions across the academic and public health practice continuum. Findings and best practices could then be shared to bolster quality across programs and strengthen relationships with the professional practice community. Furthermore, this dialog could move difficult questions such as potential over-saturation of both the job market and academic degree offerings to the forefront of the national discourse. Such introspection and appraisals from a range of stakeholders within the academic and the public health workforce communities could inform the cultivation and dissemination of deliberate, consistent, cohesive, and high-quality programs that are preparing graduates to meet identified public health needs and transition into available employment. Casting a broad net for input into such discourse is critical to allow for contextual considerations and adaptations to geographic, economic, and public health system distinctions across the country, as well as account for the culture of individual institutions and student needs, interests, and perspectives.

## Conclusions

These findings indicate that now is an opportune time for the field to provide guidance, support, and vision to the burgeoning undergraduate in public health programs. Such efforts offer promise for a learning community of academicians, practitioners, and students to enrich the public health education continuum and adopt innovative educational approaches. Ultimately, the aim of such efforts is to cultivate a public health system benefiting the workforce, the colleges, and the universities, as well as the students and ultimately the health of the public.
